# Annotations

**Published:** 1895-05-11

**Authors:** 


### Annotations.

### A Bombay M.B.

Dr. K. N. Bahadhurji has reprinted a "Memorandum" from the *Bombay Gazette*, in which he very strongly pleads the cause of a Bachelor's degree in Medicine for the native students of the Bombay University. A semi-European movement, which has been on foot for some time, and which Dr. Bahadhurji sharply criticises, insists upon a preliminary examination in arts which shall be both extensive in the range of its subjects, and thorough in its depth and practicality, as an indispensable condition of the granting of high degrees. It seems to us, however, that the most important of all the stages in this discussion has been passed over at a gallop, instead of having been studied with that seriousness which the case demands. That stage is represented by the question whether it is desirable or not desirable that any Indian University should have the power of granting the higher medical degrees at the present stage of the development of modern civilisation in India. It appears to us that there is much, very much, to be said in favour of a four or five years' residence in Great Britain, combined with study during that or a longer period at one of our universities or larger hospital medical schools, for those natives of India who aspire to the more elevated ranks of medicine. No doubt the structure of the human body is the same in India as in Great Britain; and no doubt the great facts of physiology and pathology, and the general principles of scientific medicine, are the same. But there are other factors in the case, indispensable factors, which are not the same. The scientific spirit cannot be said to encompass and inspire and prevail over the whole of modern life in our great dependency as it does here. But the essence of modern medicine is science; not science wholly, or even mainly, in its gross and material facts, but science in its spirit and temper; the inner science, without which mere facts and generalisations, however exact and full in themselves, are fruitless and dead. The spirit of science can only be breathed and lived in a prevailing scientific atmosphere. Such an atmosphere India does not, as yet, possess. Such an atmosphere prevails everywhere in this country. That is why it is so invaluable—may we not say indispensable?—for the natives of India who desire to be scholars and men of science in medicine to study in this country. Dr. Bahadhurji and his friends, if they are well advised, will reconsider the whole question from this standpoint.

### Then and Now in the Village.

The village of Haydock in Lancashire has a medical officer of health in Mr. J. E. Hayward, M.B., who has an ambition which should inspire every M.O.H., the ambition to be a veritable sanitary Providence for his people. His annual report, just published, contains many noteworthy facts. From among these we select a summarisation by Dr. Hayward of the village of Haydock "then and now." "Then" means forty years ago; "now" means last year. Forty years ago Haydock had 3,000 inhabitants; now it has 7,000. Then it had no water supply except stored rain-water water from drains, ponds, and surface wells; all liable to be contaminated by excremental and other filth; then "privy middens" of the most abominable type "abounded," and public scavenging was unknown; then offensive ponds and ditches, which were really open sewers, were common even on the sides of the main roads; the streets and roads were miry and uncleansed, and street lamps were unknown. Note the result: Epidemics of cholera, typhoid fever, and diphtheria were of comparatively common occurrence. No doubt they were piously attributed to the decrees of an inscrutable Providence. Turn now to the modern Haydock, with a population more than double what it was forty years ago. Now good drainage has banished foul ditches and open sewers; the streets are lighted with gas; the village is kept clean by public scavenging; infectious diseases are compulsorily notified; an isolation hospital is provided and used; a steam disinfector is available for the inhabitants; above all, there is an abundant supply of good water. Dr. Hayward, in short, gives us a little picture of the improvement effected in village life since the institution of local self-government. Such pictures, for the purposes of the scientific sanitarian and the future historian of the people, are invaluable. Such pictures every M.O.H., whether of town or village, ought to present annually, and to preserve in the public archives. As on a former occasion, we sincerely congratulate Dr. Hayward on his clear and interesting presentation of a most interesting sanitary story of village life.

### Oxford and its Infirmary.

At the quarterly Court of the Governors of the Radcliffe Infirmary, Oxford, held in January last, a report of a special committee on the financial state of the Infirmary was referred to the Committee of Management, with instructions to give effect to the suggestions in it and report to the Court. The report in question suggested the re-arrangement of the duties of the members of the staff, and the appointment of a house governor who should be responsible for all the details of administration. We regret to notice that at the meeting of the governors on the 24th ult. no reference was made to the important subject dealt with at the previous court. The Committee of Management appear to have taken no steps in regard to it, nor to have made any attempt to deal with the question of re-organisation, which presses for settlement. Unless or until the committee show an energetic determination to modernise the administration in the directions indicated in the report, and to actively develop the resources of the Radcliffe Infirmary, it must remain in its present unsatisfactory condition. Whether it is the enervating climate of Oxford, or whatever the cause, there can be little doubt that the Radcliffe Infirmary suffers at the present time from an absence of vigour in the administration, which seems to be fatally dilatory and slow in all its movements. We hoped Lord Dillon assumed the office of treasurer with a determination to infuse energy and vigour into the management, and we shall be glad to hear the cause of the present delay in carrying out the suggestions contained in the special committee's report. Why is it that Cambridge is so active and successful compared with Oxford?

